# Cruzipain Activates Latent TGF-β from Host Cells during *T*. *cruzi* Invasion

**DOI:** 10.1371/journal.pone.0124832

**Published:** 2015-05-04

**Authors:** Patrícia Mello Ferrão, Claudia Masini d'Avila-Levy, Tania Cremonini Araujo-Jorge, Wim Maurits Degrave, Antônio da Silva Gonçalves, Luciana Ribeiro Garzoni, Ana Paula Lima, Jean Jacques Feige, Sabine Bailly, Leila Mendonça-Lima, Mariana Caldas Waghabi

**Affiliations:** 1 Laboratório de Genômica Funcional e Bioinformática, Instituto Oswaldo Cruz, Fiocruz, Rio de Janeiro, Brazil; 2 Laboratório de Investigação Cardiovascular, Instituto Oswaldo Cruz, Fiocruz, Rio de Janeiro, Brazil; 3 Laboratório de Biologia Molecular e Doenças Endêmicas, Instituto Oswaldo Cruz, Fiocruz, Rio de Janeiro, Brazil; 4 Laboratório de Inovações em Terapias, Ensino e Bioprodutos, Instituto Oswaldo Cruz, Fiocruz, Rio de Janeiro, Brazil; 5 Laboratório de Biotecnologia e Fisiologia de Infecções Virais, Instituto Oswaldo Cruz, Fiocruz, Rio de Janeiro, Brazil; 6 Laboratório de Bioquímica e Biologia Molecular de Peptidases, Instituto de Biofisica Carlos Chagas Filho, Universidade Federal do Rio de Janeiro, Brazil; 7 INSERM, Unité 1036, Grenoble, F-38054, France; 8 Université Grenoble-Alpes—Grenoble, F-38041, France; 9 CEA, DSV,iRTSV, Laboratory of Biology of Cancer and Infection, Grenoble, F-38054, France; 10 Programa Integrado de doença de Chagas, Fiocruz, Rio de Janeiro, Brazil; Albert Einstein College of Medicine, UNITED STATES

## Abstract

Several studies indicate that the activity of cruzipain, the main lysosomal cysteine peptidase of *Trypanosoma cruzi*, contributes to parasite infectivity. In addition, the parasitic invasion process of mammalian host cells is described to be dependent on the activation of the host TGF-β signaling pathway by *T*. *cruzi*. Here, we tested the hypothesis that cruzipain could be an important activator of latent TGF-β and thereby trigger TGF-β-mediated events crucial for the development of Chagas disease. We found that live epimastigotes of *T*. *cruzi*, parasite lysates and purified cruzipain were able to activate latent TGF-β *in vitro*. This activation could be inhibited by the cysteine peptidase inhibitor Z-Phe-Ala-FMK. Moreover, transfected parasites overexpressing chagasin, a potent endogenous cruzipain inhibitor, prevented latent TGF-β activation. We also observed that *T*. *cruzi* invasion, as well as parasite intracellular growth, were inhibited by the administration of Z-Phe-Ala-FMK or anti-TGF-β neutralizing antibody to Vero cell cultures. We further demonstrated that addition of purified cruzipain enhanced the invasive activity of trypomastigotes and that this effect could be completely inhibited by addition of a neutralizing anti-TGF-β antibody. Taken together, these results demonstrate that the activities of cruzipain and TGF-β in the process of cell invasion are functionally linked. Our data suggest that cruzipain inhibition is an interesting chemotherapeutic approach for Chagas disease not only because of its trypanocidal activity, but also due to the inhibitory effect on TGF-β activation.

## Introduction

The flagellate protozoan *Trypanosoma cruzi* is the causative agent of Chagas disease, a pathology characterized by chronic inflammation associated with cardiomyopathy, which affects approximately 10 million people worldwide [1]. Cytokines such as TGF-β participate in important processes during the parasite’s life cycle. Mutant host cells lacking active TGF-β receptors display lower invasive capacity [2]. Anti- TGF-β neutralizing antibodies or chemical inhibitors of the TGF-β signaling pathway such as SB-431542 and GW-788388, inhibit cardiomyocyte invasion by *T*. *cruzi* [3–5]. Interestingly, *T*. *cruzi*, like other pathogens, can directly activate latent TGF-β present at the host cell surface and stimulate the Smad 2/3 signaling cascade, thereby allowing parasite entry into mammalian cells [3]. The molecule(s) from *T*. *cruzi* capable of activating latent TGF-β remained unknown, although some studies suggested that it could be a peptidase [3].

The main cysteine peptidase (CP) from *T*. *cruzi* is cruzipain, a papain-like endopeptidase expressed as a 57-kDa protein in all life cycle stages of the parasite, being more abundant in replicating forms and especially in the insect epimastigote stage. It is well documented to be highly homologous to other members of the papain superfamily of peptidases [6], except for its C-terminal extension, which is unique to trypanosomes [7]. Cruzipain displays dual cathepsin L and cathepsin B specificity [8], is expressed as a pre-pro-enzyme that undergoes maturation [9] and is encoded by a high number of genes (up to 130 in the Tul2 strain) giving rise to isoforms with varying degrees of similarity [10–12]. Expression has also been demonstrated to be post-transcriptionally regulated during the parasite’s life cycle [13] resulting in a complex mixture of isoforms in most of the parasite’s developmental stages, including some membrane-bound isoforms [14]. Cruzipain matures in the Golgi apparatus [15, 16] and is highly accumulated [17] and active [18] in reservosomes. Moreover, cruzipain plays vital roles during *T*. *cruzi* life cycle: it helps in the penetration of trypomastigotes into host cells [19, 20], is crucial for metacyclogenesis and intracellular development [21], participates in the development of host immune response triggered by the parasite [22] and is involved in the interaction with the insect host [23].

Cruzipain is a highly immunogenic protein and is considered one of the most attractive antigens for vaccine development, since mice immunized with cruzipain display protective immunity against parasites [24, 25]. On the other hand, cruzipain participates in the cytokine network enrolled in Chagas disease. Cytokines regulate parasite replication and immune response in infected hosts and are associated with the production of a pro-inflammatory response. Interleukin-12 triggers the production of interferon-γ- by natural killer (NK) and T cells [26]. Cruzipain induces the secretion of IL-12 by dendritic cells and favors Th1-type immune response via bradykinin B2 receptors [27]. IFN-γ is one of the major mediators of the classical macrophage activation pathway, inducing the release of nitric oxide (NO) that is responsible for intracellular parasite killing [28]. Stimulation of murine macrophages with cruzipain induces alternative activation of these cells, up-regulates arginase activity, enhances IL-10 and TGF-β production and increases *T*. *cruzi* survival [29]. NO inhibits cruzipain [30] as well as other CPs via S-nitrosylation [31]. TGF-β is able to suppress some macrophage microbicidal functions [32, 33] and is considered one of the means through which parasites convert the hostile cellular microenvironment into a favorable one, as an advantage for its survival [34, 35]. The involvement of cruzipain in TGF-β activation has not yet been demonstrated and is the aim of the present study.

TGF-β isoforms are synthesized as large biologically inactive precursors, called latent TGF-β, which are proteolytically processed to yield mature and active 25 kDa homodimers. Active TGF-β then binds to its membrane receptors, transduces intracellular signals and develops biological functions. A variety of agents and treatments are known to activate latent TGF-β, including heat, acidic pH, chaotropic agents, thrombospondin, plasmin, subtilysin-like endopeptidases, cathepsins [34, 36–38] and more recently integrins [39]. *Leishmania chagasi* and *L*. *donovani* activate latent TGF-β by a CP, cathepsin B [34, 38]. Although TGF-β activation by *T*. *cruzi* has been demonstrated [3], the identification of the enzyme(s) responsible for its activation is still lacking. Here, we tested the hypothesis that cruzipain might be an important activator of latent TGF-β and that this activation might result in a strong biological response related to the process of *T*. *cruzi* host cell invasion. Our data demonstrate that the ability of cruzipain to favor host cell invasion by *T*. *cruzi* is dependent upon TGF-βactivation.

## Results

### Epimastigote forms of *T*. *cruzi* and cruzipain activate latent TGF-β

**Fig 1:**
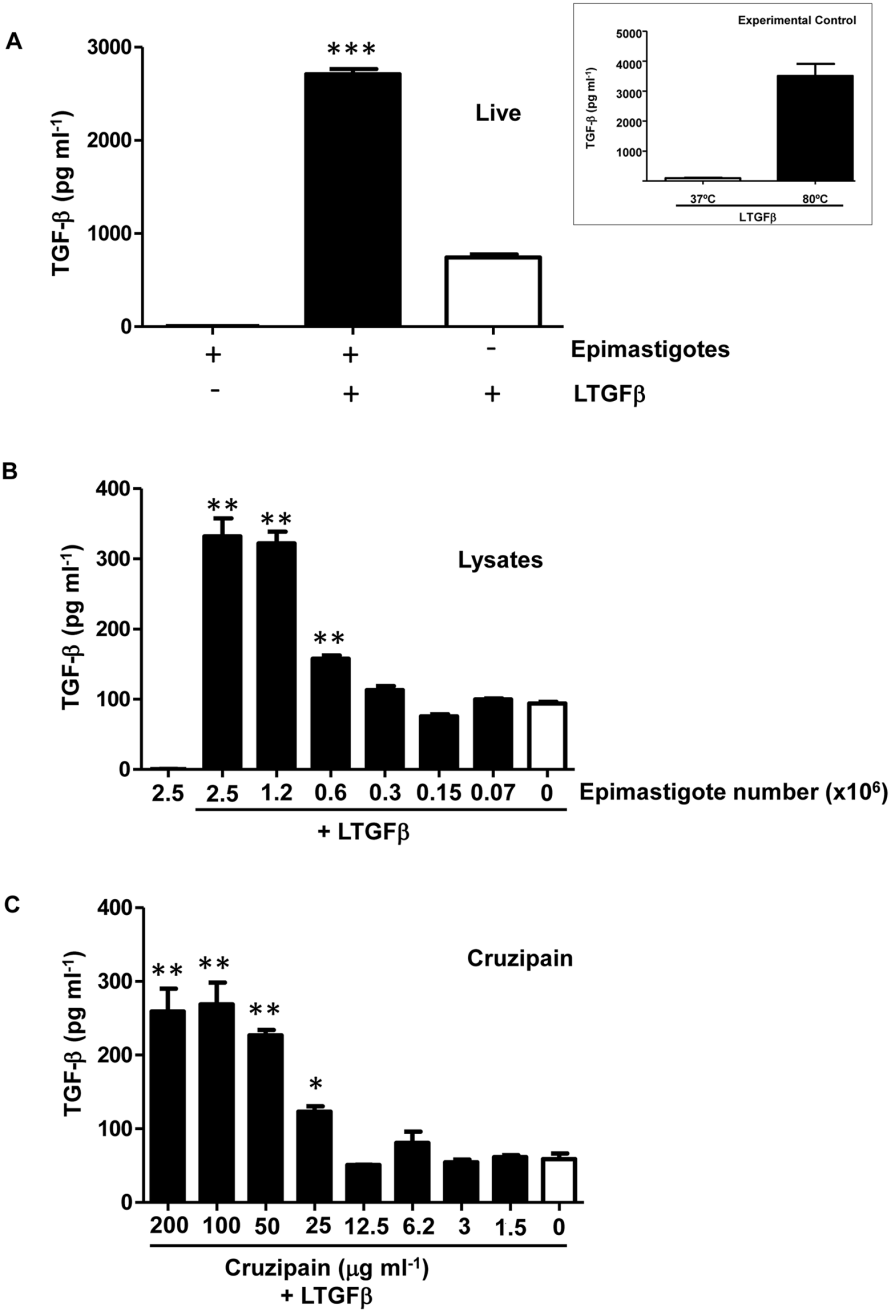
Epimastigote forms of *T*. *cruzi* and purified cruzipain activate latent TGF-β. (A) Live epimastigotes (5 × 10^6^) were incubated with latent TGF-β (100 ng) (LTGF-β) for 1 h at 28°C and the activated TGF-β was measured by ELISA. Insert: experimental controls performed in all assays (A, B and C): LTGF-β was incubated for 1 hour at 37°C (negative control) or for 10 minutes at 80°C (positive control). Levels of active TGF-β were measured in pg/ml. Activation of TGF-β was tested using different dilutions of (B) parasite lysate (equivalent to 0.07 to 2.5 × 10^6^ cells) and (C) purified cruzipain (1.5 to 200 μg ml^-1^). White bars represent recombinant latent TGF-β incubated alone for 1 h at 28°C. Data are the mean ± standard deviation (SD). (*P < 0.05, ** *P* < 0.01, *** *P* < 0.001). n = 3.

**Fig 2:**
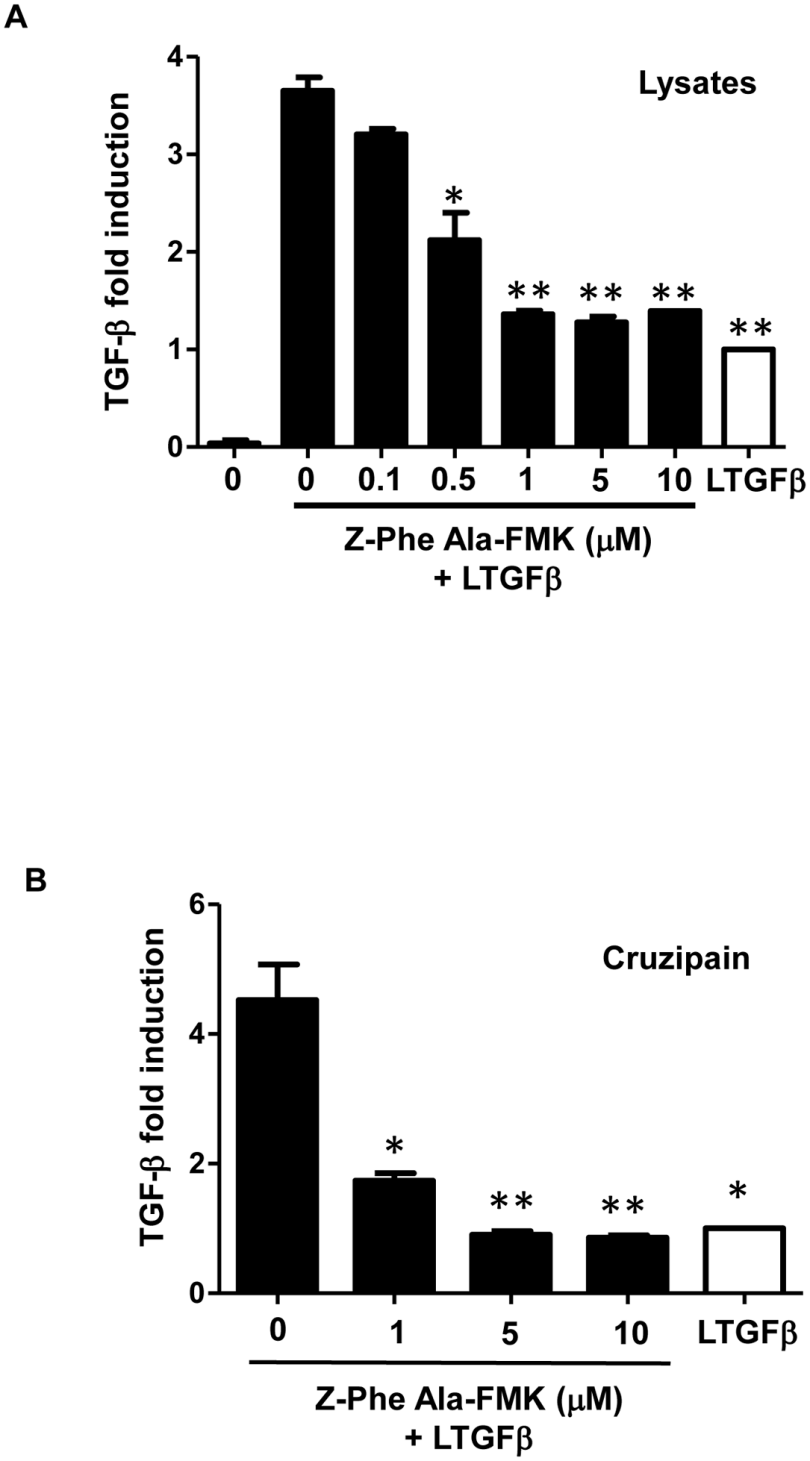
Effect of Z-Phe-Ala-FMK, a cysteine protease inhibitor, on TGF-β activation. Active TGF-β was measured by ELISA after incubation of (A) parasite lysate equivalent to 2.5 × 10^6^ epimastigotes or (B) 100 μg ml^-1^ of purified cruzipain for 1 h at 28°C with or without the cysteine peptidase inhibitor, Z-Phe-Ala-FMK, at different concentrations (0.1 to 10 μM). Results are expressed as fold induction, recombinant latent TGF-β (LTGFβ) incubated alone for 1 h at 28°C has been taken as 1 (white bars). Data are the mean ± SD. (*P < 0.05, ** *P* < 0.01). n = 3.

Since a previous study has demonstrated that both trypomastigote and amastigote forms of *T*. *cruzi* are able to activate latent TGF-β [3], we first verified whether epimastigotes could also activate latent TGF-β. Live epimastigotes were incubated with latent TGF-β and activated TGF-β was measured by ELISA, which only detects active TGF-β. As shown in Fig 1A, live epimastigotes induced TGF-β activation. In order to demonstrate that epimastigote lysates could also activate latent TGF-β, different dilutions of whole parasite extracts were tested and a dose-response was obtained (Fig 1B). Epimastigote lysates also induced TGF-β activation in a dose-dependent manner (Fig 1B). As cruzipain is one of the main peptidases expressed by *T*. *cruzi* epimastigotes, we tested if purified cruzipain could directly activate latent TGF-β *in vitro*. We found that the addition of increasing doses of purified cruzipain promoted the activation of TGF-β in a dose-dependent manner, confirming that this peptidase is capable of activating latent TGF-β in the absence of any other host or parasite factors (Fig 1C). To confirm that peptidase activity was necessary for latent TGF-β activation, we performed the assay in the presence of an irreversible inhibitor of CPs, Z-Phe-Ala-FMK, which is highly potent against cruzipain [40]. We observed that the CP inhibitor prevented the production of active TGF-β by parasite lysate (equivalent to 2.5 × 10^6^ epimastigotes) (Fig 2A) and by purified cruzipain (100 μg ml^-1^) (Fig 2B) in a dose-dependent manner, linking cruzipain activity with TGF-β activation.

### Transfected parasites overexpressing chagasin prevent TGF-β activation

**Fig 3:**
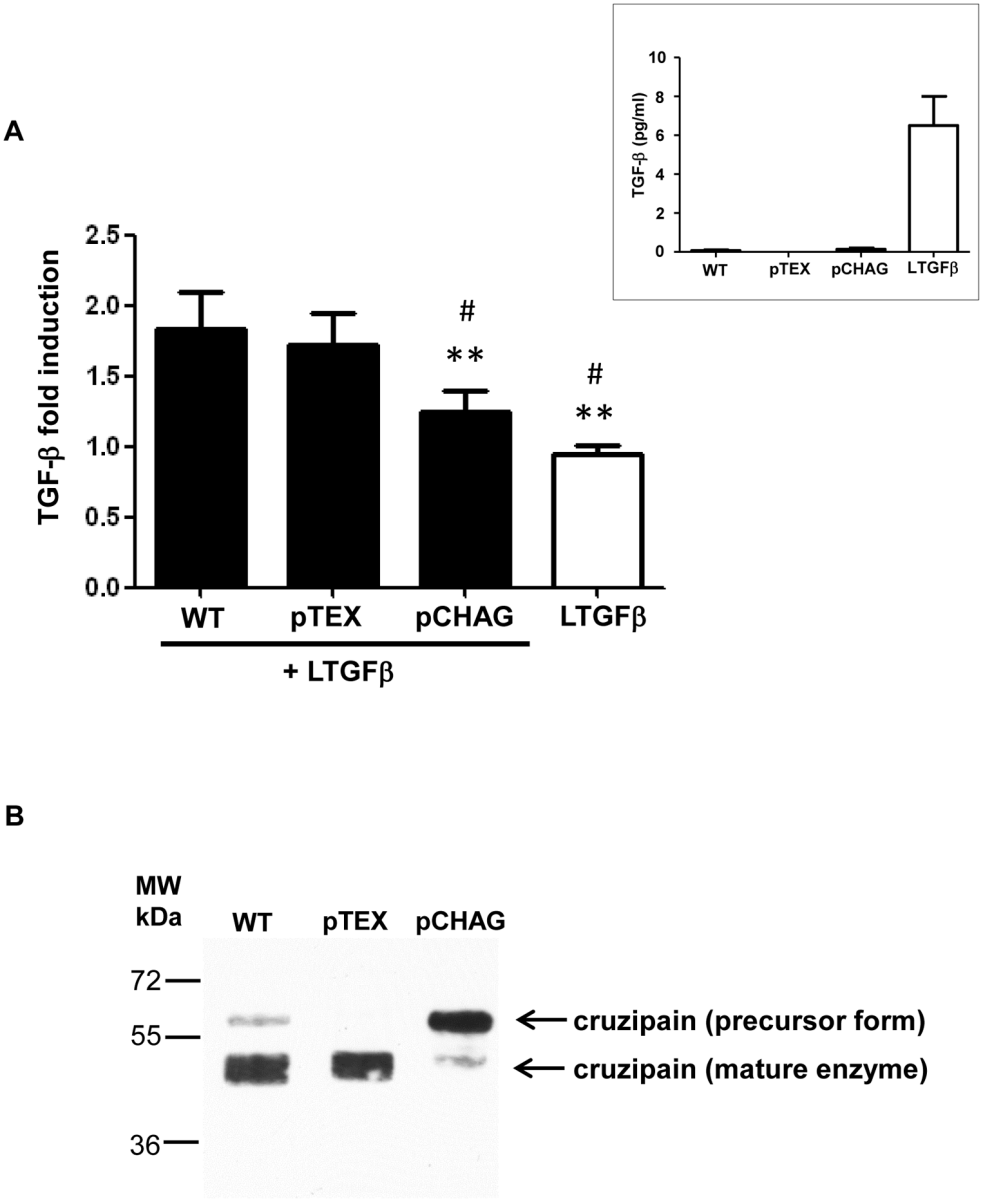
Transfected parasites overexpressing chagasin prevent TGF-β activation. (A) Live transfected Dm28c epimastigotes overexpressing chagasin (pCHAG), empty vector (pTEX) or wild type Dm28c (WT) were incubated with latent TGF-β (100 ng ml^-1^) (LTGF-β) for 1 h and the activated TGF-β was measured by ELISA. Insert: Dm28c epimastigotes that were not incubated with LTGF-β do not present significant levels of active TGF-β (pg/ml) as compared to the levels of activated LTGF-β incubated for 1h at 37°C—represented by a white bar in Fig 3A). (B) Expression pattern of cruzipain in lysates from *T*. *cruzi* Dm28c epimastigotes wild type (WT), transfected with empty vector (pTEX) or overexpressing chagasin (pCHAG) by Western blot. PageRuler Plus Prestained Protein Ladder (Thermo Scientific) was used as the molecular mass marker (MW). Results are expressed as fold induction, recombinant latent TGF-β incubated alone for 1 h at 28°C has been taken as 1. Data are the mean ± SD (*P < 0.05, ** *P* < 0.01 when compared with WT and ^#^ *P* < 0.05, ^##^ *P* < 0.01 when compared with pTEX). n = 3.

To further confirm the participation of cruzipain in TGF-β activation, we used as a tool a genetically modified *T*. *cruzi* line that overexpresses the natural cruzipain inhibitor, chagasin (pCHAG) [41]. A previous study has already demonstrated that Dm28c and Y *T*. *cruzi* isolates present similar amounts of cruzipain and similar cruzipain-chagasin molar ratios [41]. We observed that pCHAG epimastigotes displayed significantly less ability to activate latent TGF-β when compared to parasites transfected with the empty vector (pTEX) or WT parasites (Fig 3A). The presence of reduced amounts of mature cruzipain in pCHAG parasites was confirmed by western blot of parasite lysates, revealing a major band of approximately 60 kDa in pCHAG, in contrast to a major band of approximately 49 kDa in WT and pTEX (Fig 3B). As the western blot assays were performed in denaturing conditions (including boiling the samples at 100°C), we discarded the possibility that the 60 kDa band was due to the presence of chagasin-cruzipain complexes, as described elsewhere [41]. Our finding suggests that transfectants overexpressing chagasin (pCHAG) present most of their cruzipain in a precursor form (60 kDa), supporting the hypothesis that active cruzipain is required for the activation of TGF-β by *T*. *cruzi*.

### Z-Phe-Ala-FMK and anti-TGF-β antibodies inhibit parasite invasion of Vero cells

**Fig 4:**
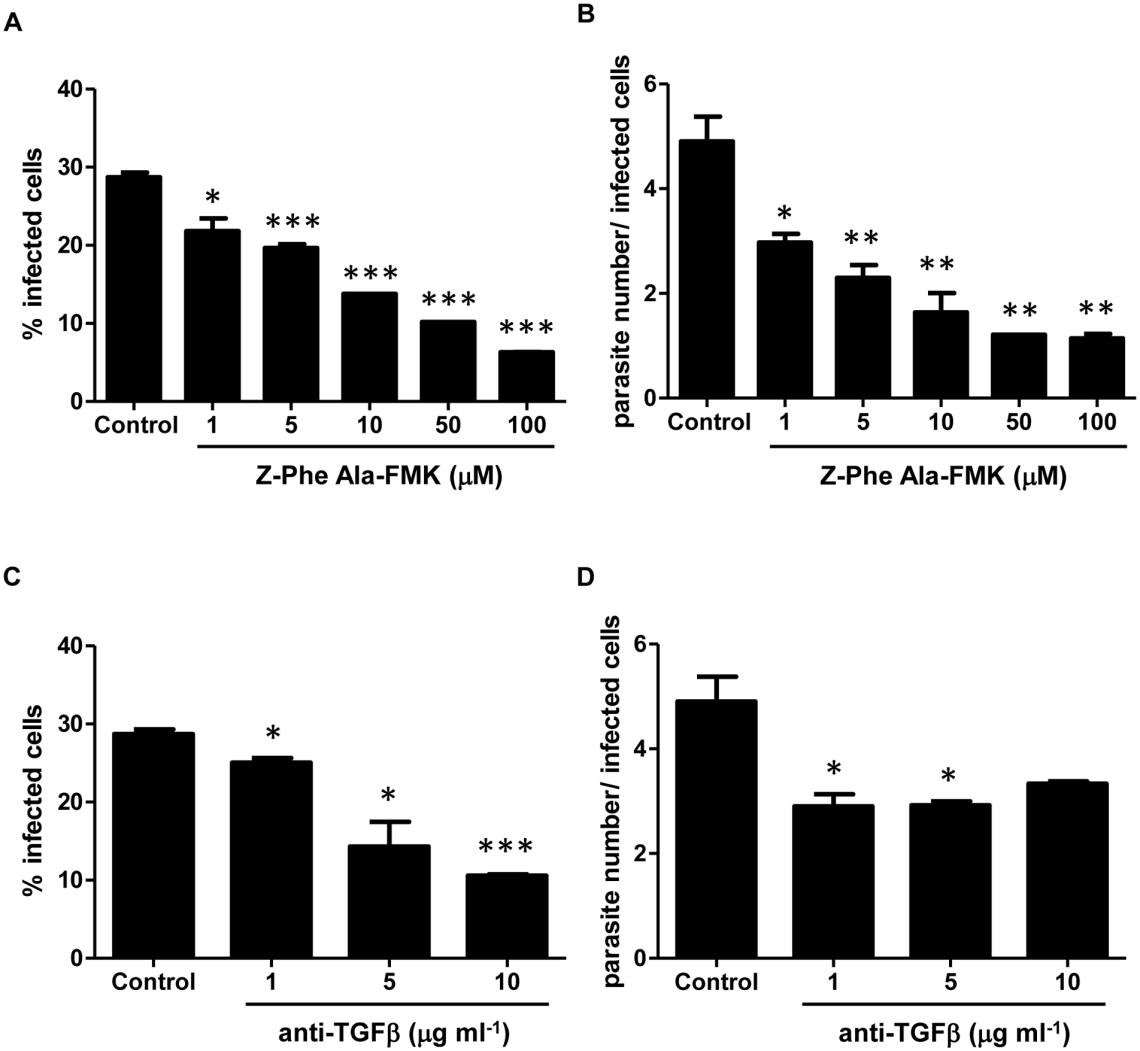
Inhibition of cysteine proteases and TGF-β activities impairs *T*. *cruzi* infection and replication. (A) Different concentrations of Z-Phe-Ala-FMK (1 to 100 μM) or (C) neutralizing anti-TGF-β antibody (1 to 10 μg ml^-1^) were added to Vero cell cultures and their effects over the processes of host cell invasion by trypomastigotes of *T*. *cruzi* (Y strain) and (B and D) intracellular parasite growth were evaluated. Results are expressed as the percentage of infected cells or as the ratio of parasite number per infected cell, both determined by counting 400 cells per slide in two distinct slides from three independent experiments. Data are the mean ± SD. (*P < 0.05, ** *P* < 0.01, *** *P* < 0.001). n = 3.

Cruzipain has been described as an important molecule in the invasion process of *T*. *cruzi* trypomastigotes into host cells [20]. We investigated whether this role could be associated to the ability of cruzipain to activate latent TGF-β. To test this hypothesis, we first analyzed the dose-response effect of either Z-Phe-Ala-FMK or neutralizing anti-TGF-β antibodies on the invasion of Vero cells by trypomastigotes. We observed that Z-Phe-Ala-FMK impaired *T*. *cruzi* invasion in a dose-dependent manner, reaching 78% of inhibition with 100 μM Z-Phe-Ala-FMK (Fig 4A). It was also observed that this compound inhibited parasite intracellular growth (Fig 4B), which could be due to a reduction in parasite invasion or due to a slower replication rate. These results are in agreement with previous observations that cruzipain inhibitors prevent host cell invasion and *T*. *cruzi* intracellular development in many *in vitro* models [19–21] and therefore confirm the validity of our model for this kind of study. The neutralization of TGF-β (1 to 10 μg ml^-1^) with anti-TGF-β antibodies also impaired both parasite invasion and replication (Fig 4C and 4D), as similarly described for *T*. *cruzi* infection on cardiomyocyte cultures [3].

### Cruzipain favors *T*. *cruzi* invasion through TGF-β activation

**Fig 5:**
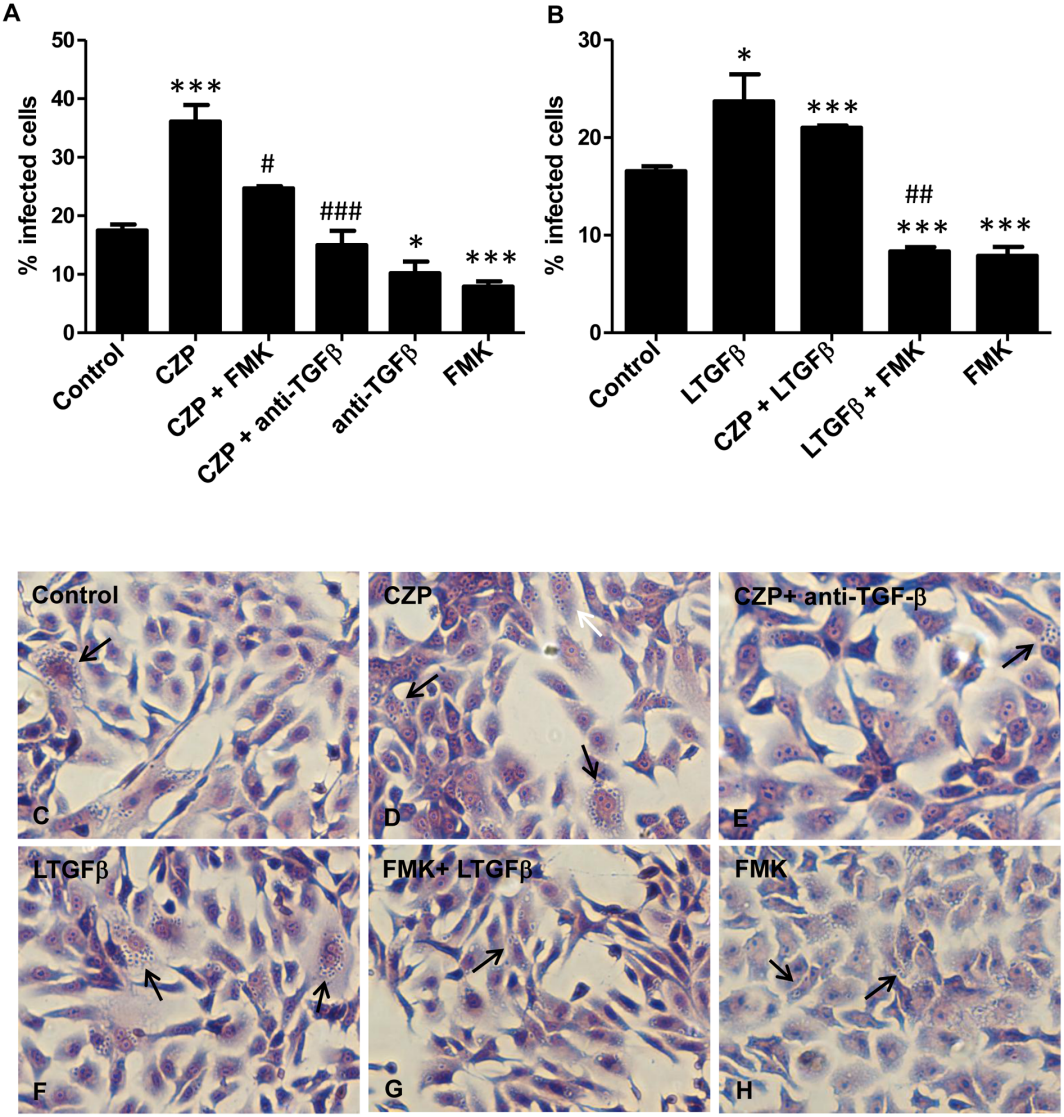
Cruzipain increases *T*. *cruzi* invasion through TGF-β activation. (A) Vero cells (1 × 10^5^ cells per well) were infected with trypomastigote forms of *T*. *cruzi* (Y strain) that were added to cultures in a mixture containing purified cruzipain (CZP, 10 μg ml^-1^) or anti-TGF-βantibodies (10 μg ml^-1^) or FMK (50 μM) or CZP (10 μg ml^-1^) in a combination with anti-TGF-β antibody (10 μg ml^-1^) or FMK (50 μM). (B) Parasites were pre-treated with different combinations of compounds before addition to Vero cell culture: trypomastigotes were treated or not with Z-Phe-Ala-FMK (FMK, 50 μM) for 30 min at 37°C and then incubated or not with recombinant latent TGF-β (LTGFβ 100 ng ml^-1^) or with LTGFβ (100 ng ml^-1^) plus CZP (10 μg ml^-1^) for 1 h at 37°C. Vero cells were then infected with pre-treated parasites. Cells were fixed 48 h post infection and stained with Giemsa. Representative images are shown (C to H). Arrows indicate infected cells. The percentage of infected cells was determined by counting 400 cells per slide in two distinct slides. Data are the mean ± SD (*P < 0.05, ** *P* < 0.01, *** *P* < 0.001 when compared with controls and ^#^ *P* < 0.05, ^##^ *P* < 0.01, ^###^ *P* < 0.001 when compared with CZP (A) and LTGFβ (B). n = 3.

To test the hypothesis that one of the mechanisms by which cruzipain mediates *T*. *cruzi* infectivity involves TGF-β activation, we added anti-TGF-β neutralizing antibodies together with purified cruzipain to Vero cell cultures and measured the percentage of infected cells. As previously described [42], addition of purified cruzipain increased *T*. *cruzi* entry into host cells (Fig 5A and 5D). This effect was completely blocked by addition of TGF-β neutralizing antibody to Vero cell cultures (Fig 5A and 5E). The enhancement in *T*. *cruzi* infection generated by addition of cruzipain to Vero cell cultures was also blocked when it was administrated together with Z-Phe-Ala-FMK (Fig 5A). When only Z-Phe-Ala-FMK was added, the percentage of infected cells decreased by half when compared to control (Fig 5A). To further demonstrate that activation of latent TGF-β by endogenous cruzipain is important for host cell invasion, we pre-incubated trypomastigote forms of *T*. *cruzi* with latent TGF-β prior to infection. We observed that pre-incubation of parasites with latent TGF-β increased the rate of infection by more than 50% (Fig 5B and 5F). When cruzipain was administrated together with latent TGF-β we did not observe a synergistic effect on *T*. *cruzi* infectivity (Fig 5B). The raise in the percentage of infected cells stimulated by incubation of parasites with latent TGF-β was impaired when parasites were treated with Z-Phe-Ala-FMK prior to incubation with latent TGF-β (62% reduction, Fig 5B and 5G), indicating a direct role of CPs on latent TGF-β activation during the parasite invasion process.

## Discussion

Over the past years, it has become clear that peptidases produced by protozoan parasites play an important role in several steps of the infection process, including: adsorption, penetration, intracellular survival, replication, differentiation, infectivity, immune evasion and nutrition [43–45]. Several studies using synthetic irreversible CP inhibitors suggested that *T*. *cruzi* infectivity and intracellular growth depend on the activity of cruzipain, the parasite’s main lysosomal CP [15, 19, 21].

Interestingly, we and others have also demonstrated that TGF-β and its downstream signaling pathways play a pivotal role not only in *T*. *cruzi* invasion but also in the completion of the parasite’s intracellular differentiation process, using both *in vitro* and *in vivo* models [2, 4, 5, 35, 46]. Moreover, our group recently demonstrated through a proteomic approach that addition of TGF-β to parasite culture medium alters the expression of several proteins in epimastigote forms, amongst them, cruzipain exhibits the most enhanced expression [47]. These convergent findings drew our attention to the possible involvement of cruzipain in the mechanism of TGF-β activation by *T*. *cruzi*.

Cruzipain is one of the most prominent *T*. *cruzi* targets for the development of drugs for Chagas disease due to its broad range of involvement in parasite’s biology. The data presented here demonstrate that cruzipain can directly activate latent TGF-β as a strategy for host cell invasion. Interestingly, it was observed that cruzipain is able to release kinin activity from secluded spaces, which could then activate the bradykinin receptor B2, facilitating parasite infection [42]. *Leishmania chagasi* and *Leishmania donovani* also activate latent TGF-β via a CP, cathepsin B [34, 38]. *Plasmodium* metalloendopeptidases can cleave latent TGF-β into its biologically active form [48] and this activity is linked to enhanced parasite survival inside host cells. Our data demonstrate that cruzipain is able to activate latent TGF-β resulting in increased infectivity by *T*. *cruzi*. Inhibition of this activation by the irreversible inhibitor, Z-Phe-Ala-FMK, strongly suggests the participation of cruzipain in this process, but does not exclude the possible participation of other CPs.

Our hypothesis is corroborated by the experiments with transfected Dm28c parasites, which provide evidence that the presence of mature cruzipain is important for TGF-β activation. In parasites overexpressing chagasin, a cruzipain inhibitor, there was a reduced level of TGF-β activation and the analysis of cruzipain expression showed that this reduction is probably because cruzipain is present mainly in a precursor form (~60 kDa). The molecular mass observed led us to the hypothesis that chagasin may be inhibiting the cleavage of the N-terminal pro-domain of cruzipain, which is necessary for production of the mature enzyme, but further studies are necessary to confirm that. Our *in vitro* results confirmed the association between the activities of cruzipain and TGF-β during *T*. *cruzi* infection, as a significant decrease in *T*. *cruzi* invasion was observed when a neutralizing anti-TGF-β antibody was added, impairing cruzipain effect on parasite infection.

Moreover, the finding that pre-incubation of parasites with Z-Phe-Ala-FMK led to a significant decrease in *T*. *cruzi* invasion, even with the posterior addition of latent TGF-β reinforces the idea that CPs, including cruzipain, are important for TGF-β activation, and therefore, to enable TGF-β to exert its positive effect on the invasion process.

The direct role of cruzipain on TGF-β activation reinforces the exploitation of this molecule as a target for the development of new therapeutic approaches for Chagas disease. TGF-β has been described to participate in processes influencing the development of myocardiopathy that occurs in Chagas disease, including: (a) the invasion process of parasites into host cells; (b) the proliferation of parasites in cardiac fibroblasts and myocytes, their differentiation into trypomastigotes and their death through apoptosis inside the host cells; (c) the regulation of inflammation and immune responses; and (d) the onset of fibrosis and heart remodeling in acute and chronic stages of the disease [revised in 49]. Thus, the impairment of TGF-β activity by inhibition of a protein unique to parasites could avoid undesirable side effects. Treatment of *T*. *cruzi*-infected mice with the cruzipain inhibitor N-methyl-piperazine-Phe-homoPhe-vinyl sulphone phenyl (K777) resulted in their effective rescue from lethal infection and in parasitological cure [40]. This effect was observed even in an immune-deficient mouse model [50, 51]. These results support the idea that anti-cruzipain compounds are effective not only due to their trypanocidal effect, but also for indirectly inhibiting different TGF-β activities, crucial for the development of Chagas disease.

## Experimental Procedures

### Parasites and sample preparation

*T*. *cruzi* epimastigotes (Y strain) were grown in liver infusion tryptose (LIT) medium supplemented with 10% fetal bovine serum (FBS) at 28°C. Lysates of epimastigotes were obtained from parasites (5 × 10^8^) from 5-day-old cultures (exponential growth phase) that were centrifuged and washed two times in phosphate buffered saline (137 mM NaCl, 2.7 mM KCl, 10 mM Na_2_HPO_4_, 2 mM KHPO_4_—PBS pH 7.4), resuspended in 0.1x PBS and submitted to four cycles of freezing—thawing (5 minutes in liquid nitrogen followed by 5 minutes at 37°C, in each cycle) and stored at -70°C. Protein concentration was determined by the RC DC method (BioRad), using bovine serum albumin as standard. The parasite strain was obtained from Fiocruz-COLPROT (Coleção de Protozoários da Fiocruz), COLPROT 106.

### Transfected parasites lines

The chagasin-encoding gene was cloned into the *Bam*HI site of the pTEX shuttle vector and transfected into *T*. *cruzi* epimastigotes from the Dm28c strain as described before [41]. Briefly, wild type and transfected parasites (empty vector, pTEX; pTEX-chagasin, pCHAG) were cultivated overnight in LIT medium containing 10% FBS and subsequently selected for 4 weeks with 200 μg ml^-1^ geneticin (G418, Life Technologies), followed by a second round of selection in medium supplemented with 800 μg ml^-1^ G418 for additional 4 weeks. The resistant population was cultivated in LIT-FCS supplemented with 800 μg ml^-1^ G418.

### Vero cells infection

Vero cells were maintained in Eagle’s medium (Sigma-Aldrich) supplemented with 10% FCS (Sigma-Aldrich), 50 μg ml^-1^ streptomycin and 1mM L-glutamine (Sigma-Aldrich). Trypomastigotes of *T*. *cruzi* (Y strain) were obtained from the supernatant of infected cultures of Vero cells, as previously described [52]. Vero cells were infected with trypomastigotes in a proportion of 10:1.

### Native and recombinant cruzipain purification and antibody production

Active cruzipain was isolated and purified from *T*. *cruzi* Dm28c epimastigotes, as previously described [53]. For recombinant protein expression, the cruzipain coding sequence was amplified from *T*. *cruzi* (Y strain) genomic DNA with primers Czp-For (5’-ATGTCTGGCTGGGCGCGTGC-3’) and Czp-Rev (5’- GAGGCGGCGATGACGGCTTT-3’). The resulting 1.4 kb fragment was purified with the Wizard PCR purification kit (Promega) and ligated with the pBAD/TOPO ThioFusion vector (Life Technologies). *Escherichia coli* TOP10 competent cells were transformed with the ligated DNA and transformants selected on LB plates containing 100 ug ml^-1^ ampicilin. The insert integrity was verified by DNA sequencing. For protein purification, a 500 mL culture of *E*. *coli* bearing the expression construct was grown to an OD_600nm_ of 0.5 and induced by the addition of 0.02% arabinose and incubated at 37°C for a further 3 h. Bacterial cells were harvested, resuspended in lysis buffer (20 mM Tris-HCl pH 7.5, 100 mM KCl, 5 mM ethylenediaminetetraacetic acid—EDTA) and lysed in a bead-beater apparatus with 0.1 mm diameter silica beads (Biospec Products). The lysate was centrifuged at 12,000 × g for 15 min at 4°C and the pellet, containing the recombinant protein in inclusion bodies, was sequentially washed three times in wash buffer (50 mM Tris-HCl pH 8.5, 0.5% Triton X-100, 5 mM EDTA, 150 mM NaCl). The final pellet was resuspended in denaturation buffer (20 mM Tris-HCl pH 7.5, 10 mM imidazole, 300 mM NaCl, 8 M urea), diluted to 4 M urea with 20 mM Tris-HCl pH 7.5, 10 mM imidazole, 300 mM NaCl and the recombinant cruzipain (rCZP) purified through a 10 ml Ni^2+^-charged chelating sepharose FF (GE) column, with elution using a linear 5–500 mM imidazole gradient. Fractions containing the purified protein were pooled and dialyzed against PBS. Polyclonal mouse anti-rCZP antibodies were obtaining by immunization of Balb/c mice (3 intraperitoneal injections of 10 μg of recombinant protein in Freund’s incomplete adjuvant at 2 weeks intervals).

This study was approved by the Commission for the Use of Laboratory Animals from FIOCRUZ (license CEUA/FIOCRUZ LW46-11).

### SDS-PAGE and immunoblotting

Total protein extracts (20 μg) were mixed with protein loading buffer (62.5 mM Tris-HCl pH 6.8, SDS 3%, Glycerol 10%, β-mercaptoethanol 1:20). The mixture was heated at 100°C for 5 min. The proteins were then resolved on 12% SDS-PAGE and transferred to nitrocellulose membranes (Hybond C, GE). Uniform sample loading and transfer were verified using Memcode reversible protein stain (Pierce). Non-specific binding sites were blocked by incubating the membranes with 5% (w/v) nonfat milk/TBS/Tween-20 0.1% overnight at 4°C. The membranes were probed with anti-cruzipain antibody (1:10.000) in 5% w/v nonfat milk, TBS/Tween-20 0.1% for 2 h and detected with appropriate secondary antibody conjugated with peroxidase (Pierce) for 1 h at room temperature. Blots were developed using Supersignal West Pico Chemiluminescent Substrate (Pierce), recorded on autoradiography film and scanned with a GS-800 scanner (BioRad) at 600 dpi resolution.

### Measurements of TGF-βactivation

Live epimastigotes from *T*. *cruzi* (Y strain), parasite lysates (5 × 10^6^) or purified cruzipain were incubated in 250 μl of PBS with 100 ng ml^-1^ of recombinant latent TGF-β (R&D) for 1 h at 28°C (live parasites) or at 37°C (lysates and cruzipain). Active TGF-β was assayed by Western blotting (as described above) or by ELISA assays (Promega), according to the manufacturer’s instructions (the minimum detectable dose of TGF-β in the ELISA assay is less than 32 pg ml^-1^). PBS incubation with the same amount of latent TGF-β was used as a control. We also tested the inhibitory effect of Z-Phe-Ala-FMK (Enzo Life Sciences) on the activation process.

### Analysis of Z-Phe-Ala-FMK and anti-TGF-β effect on parasite host-cell invasion and proliferation

Vero cells were seeded in 24-well plates (1 × 10^5^ cells per well) for 24 h. Z-Phe-Ala-FMK (1, 5, 10, 50 or 100 μM) or pan-specific TGF-β antibody (1, 5 or 10 μg ml^-1^, R&D) were added during infection of Vero cells with trypomastigote forms (Y strain) in a parasite:host cell ratio of 10:1. After 48 h, cells were washed with PBS, fixed in Bouin’s solution and stained with Giemsa. The percentage of infected cells and the number of parasites per infected cell were determined by counting at least 400 cells per slide in two distinct slides. Analysis was performed with a Nikon microscope, at 400x magnification.

### Analysis of TGF-β activation by cruzipain and its involvement in *T*. *cruzi* infection

Trypomastigotes (Y strain) were pre-treated or not with 50 μM Z-Phe-Ala-FMK for 30 min at 37°C. Parasites were then incubated with recombinant latent TGF-β (100 ng ml^-1^) for 1 h at 37°C. Vero cells seeded in 24-well plates for 24 h were then incubated with pre-treated trypomastigotes in a parasite:host cell ratio of 10:1. In parallel, Vero cells were infected with *T*. *cruzi* in a mixture containing anti-TGF-β neutralizing antibodies (10 μg ml^-1^) and exogenous cruzipain (10 μg ml^-1^). After 48 h, cells were washed with PBS, fixed in Bouin’s solution, stained with Giemsa and analyzed, as described above.

### Statistical analysis

At least three biological replicates were performed in each experiment. Differences were considered statistically significant when *P* < 0.05 comparing the mean values for control and treated cells with an Unpaired Student *t* test, calculated with GraphPadPrism 4.0 software (Graph-Pad Software Inc.).
